# I_K1_ Channel Agonist Zacopride Alleviates Cardiac Hypertrophy and Failure *via* Alterations in Calcium Dyshomeostasis and Electrical Remodeling in Rats

**DOI:** 10.3389/fphar.2019.00929

**Published:** 2019-08-26

**Authors:** Qing-Hua Liu, Xi Qiao, Li-Jun Zhang, Jin Wang, Li Zhang, Xu-Wen Zhai, Xiao-Ze Ren, Yu Li, Xiao-Na Cao, Qi-Long Feng, Ji-Min Cao, Bo-Wei Wu

**Affiliations:** ^1^Department of Pathophysiology, Shanxi Medical University, Taiyuan, China; ^2^Key Laboratory of Cellular Physiology at Shanxi Medical University, Ministry of Education, and the Department of Physiology, Shanxi Medical University, Taiyuan, China; ^3^Clinical Laboratory, Children’s Hospital of Shanxi, Taiyuan, China; ^4^Clinical Skills Teaching Simulation Hospital, Shanxi Medical University, Taiyuan, China; ^5^Department of Internal Medicine, The Hospital of Beijing Sports University, Beijing, China

**Keywords:** inward rectifier potassium channel, isoproterenol, calcium overload, cardiac remodeling, zacopride

## Abstract

Intracellular Ca^2+^ overload, prolongation of the action potential duration (APD), and downregulation of inward rectifier potassium (I_K1_) channel are hallmarks of electrical remodeling in cardiac hypertrophy and heart failure (HF). We hypothesized that enhancement of I_K1_ currents is a compensation for I_K1_ deficit and a novel modulation for cardiac Ca^2+^ homeostasis and pathological remodeling. In adult Sprague-Dawley (SD) rats *in vivo*, cardiac hypertrophy was induced by isoproterenol (Iso) injection (i.p., 3 mg/kg/d) for 3, 10, and 30 days. Neonatal rat ventricular myocytes (NRVMs) were isolated from 1 to 3 days SD rat pups and treated with 1 μmol/L Iso for 24 h *in vitro*. The effects of zacopride, a selective I_K1_/Kir2.1 channel agonist, on cardiac remodeling/hypertrophy were observed in the settings of 15 μg/kg *in vivo* and 1 μmol/L *in vitro*. After exposing to Iso for 3 days and 10 days, rat hearts showed distinct concentric hypertrophy and fibrosis and enhanced pumping function (*P* < 0.01 or *P* < 0.05), then progressed to dilatation and dysfunction post 30 days. Compared with the age-matched control, cardiomyocytes exhibited higher cytosolic Ca^2+^ (*P* < 0.01 or *P* < 0.05) and lower SR Ca^2+^ content (*P* < 0.01 or *P* < 0.05) all through 3, 10, and 30 days of Iso infusion. The expressions of Kir2.1 and SERCA2 were downregulated, while *p*-CaMKII, *p*-RyR2, and cleaved caspase-3 were upregulated. Iso-induced electrophysiological abnormalities were also manifested with resting potential (RP) depolarization (*P* < 0.01), APD prolongation (*P* < 0.01) in adult cardiomyocytes, and calcium overload in cultured NRVMs (*P* < 0.01). Zacopride treatment effectively retarded myocardial hypertrophy and fibrosis, preserved the expression of Kir2.1 and some key players in Ca^2+^ homeostasis, normalized the RP (*P* < 0.05), and abbreviated APD (*P* < 0.01), thus lowered cytosolic [Ca^2 +^]_i_ (*P* < 0.01 or *P* < 0.05). I_K1_channel blocker BaCl_2_ or chloroquine largely reversed the cardioprotection of zacopride. We conclude that cardiac electrical remodeling is concurrent with structural remodeling. By enhancing cardiac I_K1_, zacopride prevents Iso-induced electrical remodeling around intracellular Ca^2+^ overload, thereby attenuates cardiac structural disorder and dysfunction. Early electrical interventions may provide protection on cardiac remodeling.

## Introduction

Ventricular remodeling is characterized by myocardial hypertrophy and interstitial fibrosis in response to exercise or damage. It is a dynamic and time-dependent process. Physiological remodeling could improve pumping function by increasing the amount of contractile units and reducing the wall stress (Fedak et al., 2005). While maladaptive remodeling may lead to progressive ventricular dilatation, dysfunction, and even malignant arrhythmias (St John Sutton et al., 2003). Cardiac remodeling generally encompasses two components, structural remodeling, and electrical remodeling. The former exhibits hypertrophy, necrosis, apoptosis, as well as interstitial fibrosis, resulting in changes in heart size, shape, and mass (Tsukamoto et al., 2006; Ryan et al., 2007; Zhu et al., 2007; Li et al., 2009; Stewart et al., 2010). Electrical remodeling involves alterations in cardiac ion channels, exchangers, or pumps such as L-type calcium channels (LTCC), transient outward potassium channel, ATP-sensitive potassium channel (K_ATP_), inward rectifier potassium channel (I_K1_), sodium-calcium exchanger (NCX), and sodium-potassium pump (Aimond et al., 1999; Long et al., 2015). Large-scale animal and clinical trials have confirmed that β-blockers, angiotensin-converting enzyme inhibitors (ACEI), angiotensin II receptor blockers (ARB), aldosterone antagonists, and endothelin receptor antagonists avail to limit ventricular dysfunction and remodeling (reviewed by Burchfield et al., 2013). However, the mortality associated with cardiac remodeling, heart failure (HF), and malignant arrhythmias remains high. It is crucial to identify new targets and develop effective therapies.

In some cases of cardiac diseases, electrical remodeling, such as alterations in ion channels or Ca^2+^ cycling, precedes the observed depression of mechanical performance, suggesting that amelioration of electrical remodeling might be an effective therapeutic strategy against HF (Houser and Margulies, 2003; Mueller et al., 2011). K_ATP_ is reportedly involved in ventricular remodeling, and K_ATP_ channel agonists exert beneficial effects on cardiac structural remodeling and dysfunction (Lee et al., 2008; Sun et al., 2015). I_K1_ and K_ATP_ channels are both members of inward rectifier potassium (Kir) channel family and are respectively constituted by Kir2.x and Kir6.x subunits (Hibino et al., 2010). Prolongation of the action potential duration (APD) and downregulation of I_K1_ channel are well documented hallmarks of electrical remodeling in HF (Janse, 2004). Inhibition of I_K1_ also contributes to APD prolongation. Besides, I_K1_ is reduced by elevated diastolic Ca^2+^ in HF (Fauconnier et al., 2005). Therefore, I_K1_ channel is probably involved in cardiac remodeling, and I_K1_ channel agonism or up-regulation may improve cardiac structure and dysfunction.

We previously reported a selective I_K1_/Kir2.1 channel agonist, namely, zacopride. In rat ventricular myocytes, zacopride significantly enhanced I_K1_ while with no effect on other ion channels, transporters, or pumps (Liu et al., 2012, Zhai et al., 2017). Liu et al. (2016) showed that zacopride inhibited maladaptive cardiac repair following myocardial infarction (MI), and this effect was mediated by the activation of I_K1_ channel. The present study was designed to demonstrate the potential effect of zacopride on isoproterenol (Iso)-induced ventricular remodeling and to clarify the interplay between electrical remodeling and structural remodeling around Ca^2+^ dyshomeostasis.

## Materials and Methods

### Animal and Ethical Approval

Sprague-Dawley (SD) rat pups (1–3 days old, both male and female) or adult male rats (2 months old) were provided by Laboratory Animal Research Center of Shanxi Medical University (Taiyuan, China). The adult rats were housed under standard conditions, room temperature 20–24°C, humidity 40–60%, 12:12 h light dark (LD) cycles with light intensity up to 200 lux and fed standard chow and water *ad libitum*. This study was carried out in accordance with the recommendations of the guidelines for the Care and Use of Laboratory Animals (NIH, revised 2011), Ethics Committee of Shanxi Medical University. The protocol was approved by the Ethics Committee of Shanxi Medical University.

### Induction of Cardiac Hypertrophy and Failure by Isoproterenol

Cardiac hypertrophy and failure were induced by daily injection of isoproterenol (3mg/kg/d) for 3–30 days in rats *in vivo* and were evaluated by calculating the heart mass index (the ratio of heart weight/body weight or left ventricle (LV) weight/body weight), and by echocardiography, histology, confocal microscopy, patch clamp, and western blotting.

### Experimental Protocol

**Figure 1:**
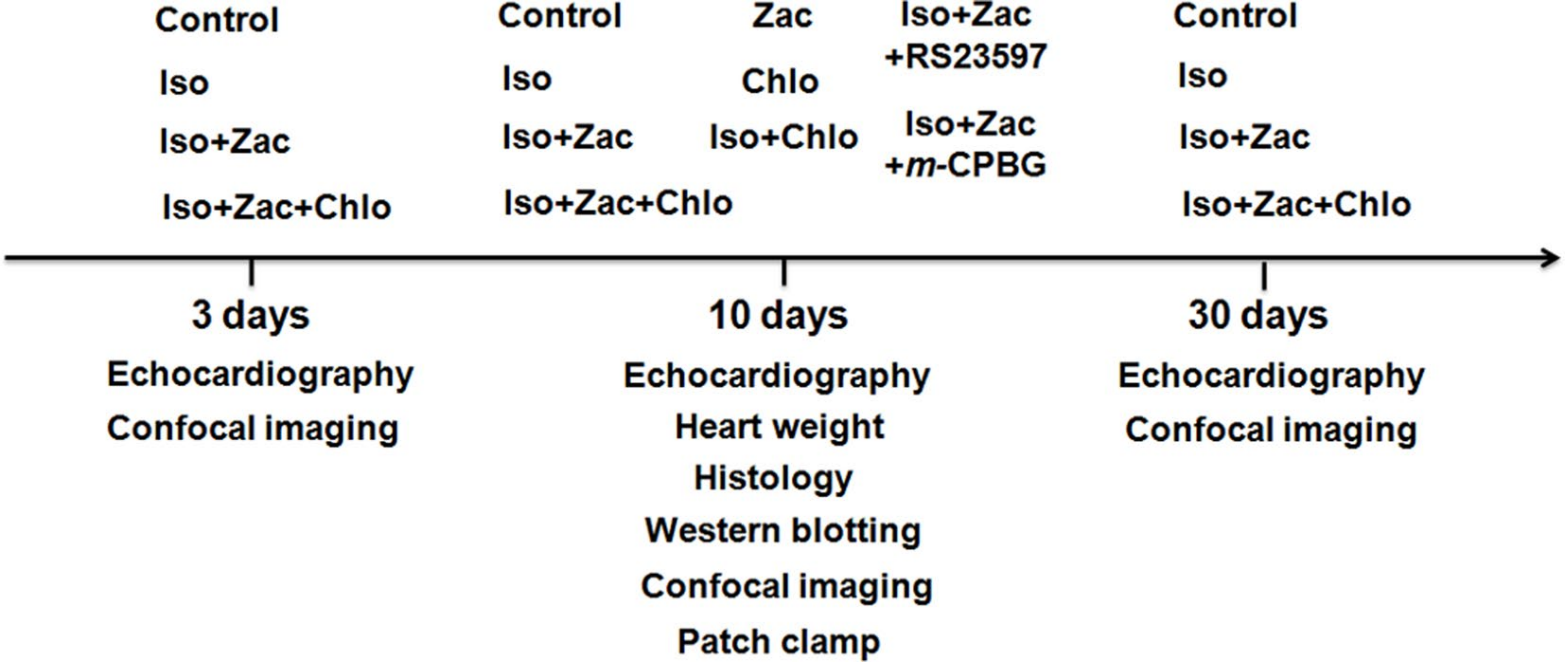
Schematic protocol of *in vivo* experiments. Iso, isoproterenol; Zac, zacopride; Chlo, chloroquine; RS23597, RS23597-190, an antagonist of 5-HT_4_ receptor. *m*-CPBG, *m*-chlorophenylbiguanide, an agonist of 5-HT_3_ receptor.

Isoproterenol (Iso, Sigma) was administered by intraperitoneal injection (i.p.) once a day for 3, 10, and 30 days, respectively, to establish temporal cardiac remodeling. An experimental protocol scheme including grouping and treatments is shown in **Figure 1**, and more information about the experiments including treatments and animal numbers is shown in **Table S1**. Pharmacological treatments were as follows: Iso (3 mg/kg/day, i.p.), zacopride (I_K1_ agonist, 15 µg/kg/day, i.p.) (Tocris, England), chloroquine (I_K1_ antagonist, 7.5 µg/kg/day, i.p.) (Sigma, USA), RS23597-190 (5-HT_4_ receptor antagonist, 0.27 mg/kg/day, i.p.) (Tocris, England), and *m*-chlorophenylbiguanide (*m*-CPBG, 5-HT_3_ receptor agonist, 0.19 mg/kg/day, i.p.) (Tocris, England). Age-matched control rats were administered with the same volume of saline. The dose of zacopride and chloroquine were applied according to our previous study (Liu et al., 2016) and preliminary experiment.

### Echocardiography

The GE Vivid 7 Pro Ultrasound System (10 S probe, probe frequency 8.0 MHz, equipped with 2D strain imaging software and EchoPAC workstation) was used in M-mode for rodent hearts. Approximate exploration angle was at 15−30°, depth at 2−3 cm, frame rate > 250/s, and maximum frame rate up to 400/s. The positioning criterion was LV long-axis section. The measured parameters included LV dimensions at end diastole (LVIDd) and end systole (LVIDs), interventricular septum thickness at end diastole (IVSd) and end systole (IVSs), LV posterior wall thickness at end diastole (LVPWd) and end systole (LVPWs), and LV ejection fraction (EF) and LV short-axis fractional shortening (FS).

### Histology

Samples of LV from all groups were fixed in 10% phosphate-buffered formalin and subjected to routine histological processing. Transverse LV sections (5 μm thick) were cut using a cryostat microtome (Leica, Wetzlar, Germany). After hematoxylin and eosin (HE) staining, the cross-sectional area of myofibers was measured using a microscope (Olympus, Tokyo, Japan) under a high-powered field (HPF) (×250 magnification). Fibrosis was evaluated by Masson’s trichrome staining, and the collagen content in the interstitial space was estimated by analyzing the images of each group. Total collagen area was calculated and expressed as percent of total ventricular area (Benjamin et al., 1989).

### Western Blotting

Proteins from LV samples were loaded (40 μg per lane) on 5−15% acrylamide gels. After electrophoretic transfer and incubation with 5% non-fat milk in Tris-buffered saline (TBS), the nitrocellulose membranes were incubated overnight at 4°C with target-protein antibodies. Standard western blotting were performed using respective antibodies to quantify the relative levels of Kir2.1 (mouse monoclonal anti-Kir2.1, dilution 1:1,000, Sigma; or rabbit monoclonal anti-Kir2.1, 1:1,000, Abcam), CaMKII and phosphorylated CaMKII (rabbit monoclonal anti-CaMKII, 1:1,000, Cell Signaling), SERCA2 (rabbit polyclonal anti-SERCA2, 1:1,000, Cell Signaling), RyR2-phospho S2808 or total RyR2 (rabbit polyclonal anti-(*p*)-RyR2, 1:1,000, Abcam), cleaved caspase 3 (rabbit monoclonal anti-c-caspase 3, 1:1,000, Cell Signaling). The GAPDH (rabbit monoclonal anti-GAPDH, 1: 2,000, Sigma) was used as the loading control in each case. Quantification of bands was executed by ImageJ and Image Lab.

### Isolation of Adult Rat Ventricular Myocytes (ARVMs)

LV myocytes from adult rats in control, Iso, Iso+Zac, and Iso+Zac+Chlo groups were respectively isolated using an enzymatic dissociation procedure. In brief, after anesthesia (sodium pentobarbital, 65 mg/kg, *i.p.*), the heart was quickly harvested and placed into chilled (4°C), oxygenated (100% O_2_), and Ca^2+^-free Tyrode’s solution and then was mounted onto a Langendorff retrograde perfusion apparatus *via* the aorta with a perfusion pressure of 80-cm H_2_O. The composition of Tyrode’s solution was (in mmol/L): NaCl 135.0, KCl 5.4, CaCl_2_ 1.8, MgCl_2_ 1.0, NaH_2_PO_4_ 0.33, HEPES 10.0, and glucose 10.0 (pH 7.3−7.4 adjusted with NaOH). The heart was perfused first with oxygenated (100% O_2_) and Ca^2+^-free Tyrode’s solution at 37°C for 10 min, and then perfused with enzyme-containing Tyrode’s solution for about 20 min until the tissue was adequately digested. The enzyme-containing Tyrode’s solution was composed of (in mmol/L) NaCl 125.0, KCl 5.4, MgCl_2_ 1.0, NaH_2_PO_4_ 0.33, HEPES 10.0, glucose 10.0, taurine 20.0, and 5.0−8.0 mg/50 ml collagenase P (Roche, Switzerland). LV myocytes was then separated and stored in Krebs buffer (KB) solution at room temperature (25°C) at least 4 hours before use. The KB solution contained (in mmol/L): KOH 85.0, L-glutamic acid 50.0, KCl 30.0, MgCl_2_ 1.0, KH_2_PO_4_ 30.0, glucose 10.0, taurine 20.0, HEPES 10.0, and EGTA 0.5. The pH was adjusted to 7.4 with KOH.

### Measurements of Cytosolic Ca^2+^ and SR Ca^2+^ Levels in ARVMs

The extracellular Ca^2+^ of ARVMs was recalcificated gradiently to 1.0 mmol/L with modified Tyrode’s solution. Cells from different groups were incubated with 5 μmol/L Fluo-4 AM (cytosolic Ca^2+^ indicator, Dojindo, Japan) and 5 μmol/L Fluo-5N/AM (SR Ca^2+^ indicator, Invitrogen, USA) respectively in fresh Tyrode’s solution (1.0 mmol/L Ca^2+^) supplemented with BSA (0.5%) at 37°C for 45 min. Unincorporated Fluo-4 or Fluo-5N was removed by washing myocytes thrice in modified Tyrode’s solution. The average intensity of Ca^2+^ fluorescence in cardiomyocytes was recorded using FV1000 laser confocal scanning microscope (Olympus, Japan).

### Patch Clamp to Record Transmembrane Potential of Cardiomyocytes

To measure the resting potential (RP) and action potential (AP) of LV myocytes, Tyrode’s solution was used as the bath solution. The pipette solution contained (in mmol/L) KCl 150.0, MgCl_2_ 1.0, EGTA 5.0, HEPES 5.0, and ATP-K_2_ 3.0; pH was adjusted to 7.3 with KOH. Cells were superfused with bath solution at 36°C, and the perfusion flow rate was at 2 ml/min. Current clamp mode of whole-cell configuration was performed using Axopatch-200B patch clamp amplifier (Axon Instrument, USA). Patch electrodes were made by a two-stage vertical microelectrode puller (PP-83, Narishge Scientific Instrument, Japan) with resistance of 2−5 MΩ. The pClampex 8.2 program (Axon Instrument, USA) was utilized to produce clamping commands. The RP results were corrected for the calculated junction potential (–8 mV).

### Isolation of Neonatal Rat Ventricular Myocytes (NRVMs)

NRVMs from 80 neonatal SD rats were isolated and cultured as previously described (Chlopcíková et al., 2001). Briefly, a combination of trypsin (0.08%, Sigma) and collagenase II (0.04%, Sigma) was used to dissociate the dissected pieces of ventricular tissues into single cells. The tissue pieces in enzyme solution were stirred gently for 6 min. The cell suspension was collected in 20% fetal bovine serum (FBS, Gibco), and the remaining tissue fragments were further digested by fresh enzyme solution. After 5−8 digestion cycles, all the supernatants containing isolated cells were collected and centrifuged at 4°C, 600 rpm for 6 min; washed once; and resuspended in DMEM culture medium containing 15% FBS. Non-myocytes were removed by differential adhesiveness, and cardiomyocytes were plated at a density of 2 × 10^5^ viable cells in culture medium supplemented with 5-bromo-2-deoxyuridine (0.1 mmol/L, Sigma). Cultured neonatal cardiomyocytes were randomly separated into six groups: control, Iso (1 μmol/L), zacopride (1 μmol/L), Iso+zacopride, Iso+zacopride+BaCl_2_ (1 μmol/L), and Iso+zacopride +chloroquine (0.3 μmol/L). All the NRVMs were incubated for 24 h after drug treatment for further study.

### Confocal Microscopy to Measure Intracellular [Ca^2+^] of NRVMs

NRVMs were incubated with 5 μmol/L Fluo-4 AM (Dojindo, Japan) in DMEM solution containing 0.5% BSA and 1 mM CaCl_2_ at 37°C for 0.5 h. Unincorporated Fluo-4 AM was removed by washing myocytes twice in PBS. The cell surface area and the intensity of [Ca^2+^]_i_ fluorescence in cardiomyocytes were recorded using FV1000 laser confocal scanning microscope (Olympus, Japan).

### Flow Cytometry to Measure Apoptosis of NRVMs

Flow cytometry was performed with propidium iodide (PI) and fluorescein isothiocyanate (FITC)–labeled Annexin V (KeyGEN Biotech, Nanjing, China). In brief, the NRVMs were treated for 24 h with different drugs as described in the grouping, then were harvested, rinsed twice with cold PBS, resuspended in binding buffer at the density of 1 × 10^6^ cells/ml, and incubated with 5 μmol/L FITC-Annexin V and 5 μmol/L PI. Cells were gently vortexed and incubated in the dark for 15 min at room temperature. Flow cytometry was performed within 1 h using a FC500 Flow Cytometer (Coulter, Beckman, Palo Alto, CA, USA).

### Statistical Analysis

All statistical analyses were performed using SPSS statistics software version 17.0 (IBM Corp, Chicago, IL, United States). Data were expressed as the mean ± standard error (SEM). Quantitative data were analyzed by one-way ANOVA. Multiple comparisons were performed using the least significant different test. Differences in mortality among groups were analyzed using chi-square test. *P* < 0.05 was considered statistically significant.

## Results

### The Mortality Rate of Iso-Modeled Rats

As shown in **Table 1**, daily Iso infusion for 3 d, 10 d, and 30 d led to sudden death in some rats. The mortalities were 40%, 41.7% (*P* < 0.01 *vs*. control), and 50% (*P* < 0.05 *vs*. control), respectively. In zacopride-treated groups, the mortalities were 16.7%, 23.8%, and 30.0% for 3, 10, and 30 days, respectively, but did not reach a statistical significance compared with the Iso group. Co-application of zacopride and chloroquine (I_K1_ channel blocker) increased the mortalities respectively to 30% (3 days), 37.5% (10 days, *P* < 0.01 *vs*. control), and 58.3% (30 days, *P* < 0.05 *vs*. control). All control rats, rats treated with zacopride alone or chloroquine alone, survived in the entire period of the experiments. These results indicate that 15 µg/kg zacopride and 7.5 µg/kg chloroquine *per se* had no significant toxicity to the rats.

**Table 1:** The mortalities of rats in a time-course study *in vivo*.

Group	Total rats (n)	Mortality (%)	Survival rats (n)
**3 days**			
Control	5	0	5
Iso	10	40	6
Iso+Zac	7	16.7	6
Iso+Zac+Chlo	10	30.0	7
**10 days**			
Control	15	0	15
Zac	15	0	15
Chlo	12	0	12
Iso	24	41.7**	14
Iso+Zac	21	23.8	16
Iso+Chlo	18	44.4**	10
Iso+Zac+Chlo	24	37.5**	15
Iso+Zac+RS23597	6	0	6
Iso+Zac+*m*-CPBG	6	16.7	5
30 days*			
Control	6	0	6
Iso	12	50.0*	6
Iso+Zac	10	30.0	7
Iso+Zac+Chlo	12	58.3*	5

*P < 0.05, **P < 0.01 vs. control.

### Morphological Features of Iso-Induced Cardiac Hypertrophy *in Vivo*

**Figure 2:**
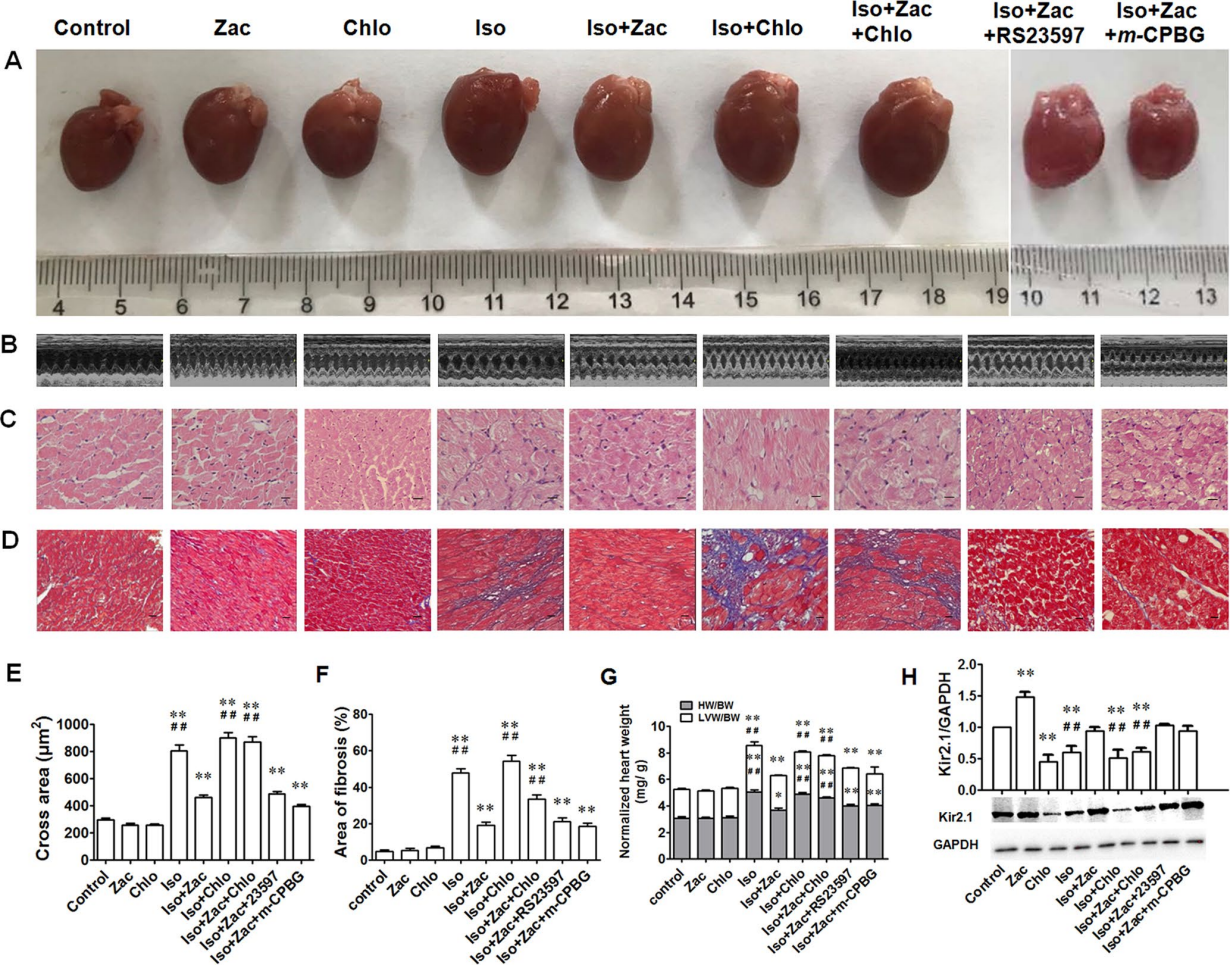
Cardiac remodeling induced by 10 days of Iso exposure in rats *in vivo*. **(A)** The gross morphology of the whole hearts. **(B)** Representative echocardiographic images from each of the corresponding hearts shown in **(A)**. **(C)** HE staining of transverse LV sections (250×). Scale bars = 50 µm. **(D)** Masson’s trichrome staining showing collagen deposition in rat LV (100 ×). Cardiomyocytes and collagen fibers were stained as red and blue, respectively. Scale bars = 100 µm. **(E)** Statistical results of the cross sectional area of myofiber in different groups. N = 50 in each group. **(F)** Statistical results of fibrotic area which was expressed as the percentage of total area in each field, n = 5 in each group. **(G)** The heart/LV mass index. **(H)** The expression levels of Kir2.1 channel protein. Iso, isoproterenol; Zac, zacopride; Chlo, chloroquine; RS23597, RS23597-190; *m*-CPBG, *m*-chlorophenylbiguanide. Data are presented as mean ± SEM. *P < 0.05, **P < 0.01 compared with control. ^##^ *P* < 0.01 compared with Iso+Zac.

The gross morphology of the whole heart (**Figure 2A**) revealed cardiac enlargement 10 days after daily Iso exposure. Echocardiographic detection demonstrated distinct characteristics of concentric hypertrophy and enhanced pumping function (**Figure 2B** and **Table 2**). To conform the results obtained from echocardiography, histological examination was performed by HE (**Figure 2C**) and Masson’s trichrome staining (**Figure 2D**). As shown in **Figures 2C and E**, compared with controls, the cardiac myofibers in Iso-treated rats are disorganized and hypertrophic, with certain degree of cell necrosis and relatively light staining of the cytoplasm. In zacopride-treated rats, cardiac myofibers are better arranged and with normal size. I_K1_ channel blocker chloroquine abolished these protective effects from zacopride as shown by worsened manifestation, indicating that the anti-remodeling effect of zacopride is mediated by the activation of I_K1_ channels. After 10 days of Iso infusion, rat hearts exhibited significant fibrosis, validated by increased collagen deposition (**Figures 2D, F** *P* < 0.01 *vs*. control). Zacopride treatment dramatically attenuated the fibrosis (*P* < 0.01), and this effect was largely abolished by chloroquine (*P* < 0.01). Iso-induced cardiac hypertrophy was further measured by heart mass index. The whole-heart weight and LV weight, normalized to body weights (**Figure 2G**), were greater in Iso-treated rats than controls (*P* < 0.01 *vs*. control). Zacopride treatment effectively prevented Iso-induced cardiac hypertrophy (*P* < 0.01), and the effect was attenuated by chloroquine (*P* < 0.01).

**Table 2:** Echocardiographic parameters in Iso-induced cardiac remodeling (mean ± SEM).

	n	IVSd (mm)	IVSs (mm)	LVIDd (mm)	LVIDs (mm)	LVPWd (mm)	LVPWs (mm)	EF(%)	FS (%)
**3 days**									
Control	5	1.54 ± 0.11	2.63 ± 0.18	5.11 ± 0.30	2.95 ± 0.28	1.79 ± 0.08	2.52 ± 0.18	79.2 ± 2.9	42.6 ± 3.3
Iso	5	2.47 ± 0.10**^##^	3.84 ± 0.30*	4.11 ± 0.34*^#^	1.84 ± 0.14**	2.00 ± 0.16	2.87 ± 0.08	88.4 ± 3.0*	54.2 ± 4.4
Iso+Zac	5	1.79 ± 0.06	3.29 ± 0.20	5.12 ± 0.14	2.17 ± 0.16*	1.82 ± 0.07	3.28 ± 0.25	90.6 ± 1.7*	57.2 ± 3.3*
Iso+Zac+Chlo	5	2.25 ± 0.14**^##^	3.45 ± 0.29*	4.35 ± 0.32	1.88 ± 0.21**	2.06 ± 0.10	3.16 ± 0.43	89.4 ± 3.8*	56.2 ± 5.1*
**10 days**									
Control	6	1.50 ± 0.06	2.50 ± 0.16	5.16 ± 0.28	2.91 ± 0.10	1.61 ± 0.12	2.45 ± 0.16	80.0 ± 1.9	43.2 ± 2.0
Zac	6	1.70 ± 0.08	2.84 ± 0.15	5.43 ± 0.32	2.91 ± 0.23	1.77 ± 0.11	2.91 ± 0.12	81.8 ± 2.9	46.0 ± 3.5
Chlo	6	1.56 ± 0.09	2.73 ± 0.22	5.05 ± 0.28	2.45 ± 0.24	1.82 ± 0.08	2.93 ± 0.18	86.3 ± 3.4	51.7 ± 4.3
Iso	7	2.14 ± 0.07**^##^	3.61 ± 0.15**^#^	5.32 ± 0.33	2.32 ± 0.26	1.91 ± 0.11*	2.84 ± 0.25	90.1 ± 2.0**	56.4 ± 3.1**
Iso+Zac	6	1.66 ± 0.03	2.97 ± 0.12	5.31 ± 0.34	2.36 ± 0.22	1.72 ± 0.09	2.93 ± 0.15	90.0 ± 1.5**	55.7 ± 12.6*
Iso+Chlo	5	2.15 ± 0.14**^##^	3.93 ± 0.28**^##^	5.14 ± 0.39	1.94 ± 0.34*	1.86 ± 0.12	3.05 ± 0.23*	92.6 ± 2.7**	62.2 ± 5.2**
Iso+Zac+Chlo	6	2.07 ± 0.10**^##^	3.68 ± 0.20**^#^	4.91 ± 0.63	2.25 ± 0.39	1.99 ± 0.10*	3.01 ± 0.13*	89.2 ± 2.4**	54.8 ± 3.5*
Iso+Zac +RS23597	5	1.78 ± 0.04*	3.24 ± 0.16*	5.22 ± 0.21	2.57 ± 0.25	1.67 ± 0.16	2.54 ± 0.10	86.8 ± 2.2	51.0 ± 3.1
Iso+Zac +*m*-CPBG	5	1.73 ± 0.10	3.39 ± 0.24**	4.85 ± 0.25	2.17 ± 0.16	1.77 ± 0.08	2.77 ± 0.13	90.2 ± 1.0**	55.4 ± 1.5*
**30 days**									
Control	6	1.78 ± 0.05	2.52 ± 0.12	5.03 ± 0.35	2.84 ± 0.26	1.86 ± 0.18	2.89 ± 0.20	80.3 ± 2.0	43.7 ± 2.3
Iso	6	1.43 ± 0.22	2.44 ± 0.14	6.41 ± 0.37*	4.35 ± 0.23**^#^	1.78 ± 0.07	2.47 ± 0.10	66.2 ± 1.2**^##^	32.0 ± 1.1**^##^
Iso+Zac	7	1.72 ± 0.10	2.50 ± 0.13	5.48 ± 0.31	3.32 ± 0.19	1.75 ± 0.15	2.54 ± 0.23	75.4 ± 1.1	39.4 ± 1.3
Iso+Zac+Chlo	5	1.59 ± 0.10	2.76 ± 0.19	6.57 ± 0.42**^#^	4.46 ± 0.42**^##^	1.89 ± 0.13	2.62 ± 0.19	66.8 ± 3.4**^#^	32.4 ± 2.2**^#^

Iso, isoproterenol; Zac, zacopride; Chlo, chloroquine; m-CPBG, m-chlorophenylbiguanide. The dose of zacopride is 15 µg/kg. LVIDd, left ventricular dimension in end diastole; LVIDs, left ventricular dimension in end systole; IVSd, interventricular septum end-diastolic thickness; IVSs, interventricular septum end-systolic thickness; EF, ejection fraction; FS, fractional shortening.*P < 0.05, **P < 0.01 vs. control. ^#^P < 0.05, ^##^P < 0.01 vs. Iso+ Zac.

Zacopride (15 µg/kg) or chloroquine (7.5 µg/kg) *per se* had no significant effects on cardiac structure or function. Because zacopride is also a known 5-HT_4_ receptor agonist and 5-HT_3_ receptor antagonist, we examined whether 5-HT receptors are involved in the anti-remodeling effect using pharmacological tools, 5-HT_4_ receptor antagonist RS23597-190, and 5-HT_3_ receptor agonist *m*-CPBG. Results showed that RS23597-190 and *m*-CPBG could not counteract the effects of zacopride on cardiac remodeling including changes of hypertrophy and fibrosis (**Table 2**, **Figures 2A–G**), suggesting that the protective effects of zacopride on LV remodeling is mediated by I_K1_ channel but not by 5-HT receptors.

### The Expression of Kir2.1 Channel Protein in Iso-Induced Hypertrophic LV

The native I_K1_ channels in the heart are assembled by Kir2.1 (KCNJ2), Kir2.2 (KCNJ12), and Kir2.3 (KCNJ4) channels. We have proven that Kir2.1 is the dominant isoform in rat ventricles and zacopride is a selective Kir2.1 channel agonist (Zhang et al., 2013). Compared with control, zacopride treatment (15 µg/kg/d) alone for 10 days upregulated Kir2.1 channel protein by 48.6% (*P* < 0.01), while chloroquine (7.5 µg/kg/d) treatment alone for 10 days decreased Kir2.1 channel protein level by 44.4% (*P* < 0.01) (**Figure 2H**). Consistently, zacopride prevented Iso-induced I_K1_ inhibition (*P* < 0.01), and this effect could be reversed by chloroquine (*P* < 0.01). Coapplication of RS23597-190 (5-HT_4_R antagonist) or *m*-CPBG (5-HT_3_R agonist) could not counteract the effect of zacopride on Kir2.1 expression (**Figure 2H**).

### The Temporal Significance of Zacopride on Iso-Induced Cardiac Remodeling, Dysfunction, and Ca^2+^ Dyshomeostasis

#### Echocardiography Revealed the Protective Effects of Zacopride on Iso-Induced Cardiac Structural Remodeling and Dysfunction *in Vivo*

**Figure 3:**
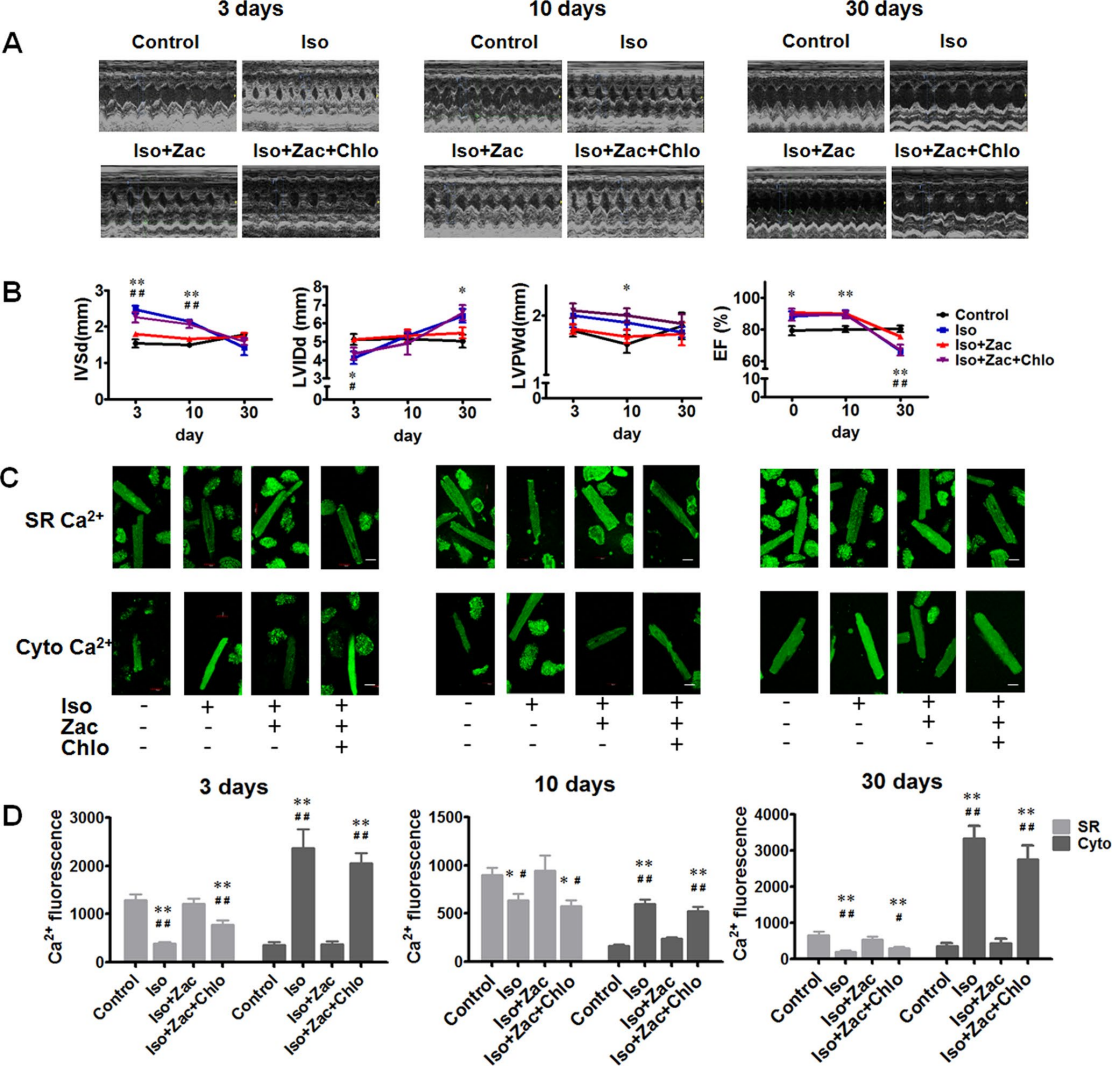
Time courses of structural remodeling and electrical remodeling 3 days, 10 days, and 30 days post-Iso infusion. **(A)** Representative echocardiographic images of the corresponding hearts. **(B)** Time courses of IVSd, LVIDd, LVPWd, and EF changes post-Iso toxication. *P < 0.05, **P < 0.01 for Iso compared with age-matched control. ^#^*P* < 0.05, ^##^*P* < 0.01 for Iso compared with age-matched Iso+Zac group. **(C)** Cytosolic Ca^2+^ and SR Ca^2+^ fluorescences measured using laser scanning confocal microscopy. Upper row, Fluo-5N cellular distribution indicating SR Ca^2+^. Lower row, Fluo-4 cellular distribution indicating cytosolic Ca^2+^. Scale bars = 20 µm. **(D)** Statistical summary of cytosolic Ca^2+^ and SR Ca^2+^ levels of 3 days, 10 days, and 30 days post-Iso exposure, respectively. SR, sarcoplasmic reticulum. LVIDd, left ventricular dimension in end diastole. IVSd, interventricular septum end-diastolic thickness. LVPWd, LV posterior wall thickness at end diastole; EF, ejection fraction; Iso, isoproterenol; Zac, zacopride; Chlo, chloroquine; RS23597, RS23597-190; *m*-CPBG, *m*-chlorophenylbiguanide. Data are presented as mean ± SEM. *P < 0.05, **P < 0.01 compared with age-matched control. ^#^*P* < 0.05, ^##^*P* < 0.01 compared with age-matched Iso+Zac.

To elucidate the precise relationship between electrical remodeling and structural remodeling in a temporal sense, we observed the development of cardiac remodeling in the period of 3, 10, and 30 days of Iso infusion *in vivo*. Post 3 days of Iso infusion, IVSd, and IVSs were increased (*P* < 0.01); LVIDd (*P* < 0.05) and LVIDs (*P* < 0.01) were reduced compared with age-matched control rats (**Table 2** and **Figures 3A−B**). Ten days after Iso treatment, IVSd (*P* < 0.01), IVSs (*P* < 0.01), and LVPWd (*P* < 0.05) were increased, while LVIDs and LVIDd had no significant differences compared with age-matched control. LVEF and LVFS were increased both in 3 days (*P* < 0.05) and 10 days (*P* < 0.01) of Iso groups compared with age-matched controls. These results indicated that Iso-induced cardiac remodeling occurred much early and was characterized by concentric hypertrophy and enhanced pumping function. Zacopride treatment prevented the thickening of interventricular septum and the decrease of LV volume (*P* < 0.01 or *P* < 0.05). 30 days after Iso treatment, LVIDd (*P* < 0.05) and LVIDs (*P* < 0.01) were significantly increased; LVEF and LVFS (*P* < 0.01) were decreased compared with age-matched control. The thickness of IVS was decreased compared with that in 3 and 10 days of Iso groups (*P* < 0.01). Collectively, these results indicated that longer (30 days) Iso exposure led to progression of cardiac pathological remodeling, dysfunctions, and even failure. Zacopride treatment prevented LV chamber dilatation (*P* < 0.05) and preserved the systolic function (*P* < 0.01). These effects were largely reversed by I_K1_ channel antagonist chloroquine (*P* < 0.01 or *P* < 0.05).

#### Confocal Microscopy Exhibited the Beneficial Effect of Zacopride on Iso-Induced Ca^2+^ Dyshomeostasis in Cardiomyocytes *in Vitro*

It is known that more than 90% of the Ca^2+^ is cycled between the cytosol and the SR in the rat (Bers, 2002). To observe the Ca^2+^ homeostasis in cardiomyocytes, we used Fluo-4 AM to quantify the cytosolic [Ca^2+^] and Fluo-5N AM as SR Ca^2+^ indicator. **Figure 3C** shows representative confocal images for cytosolic Ca^2+^ and SR Ca^2+^ fluorescence. Compared with the age-matched control, cardiomyocytes from the Iso-treated rat hearts exhibited higher cytosolic Ca^2+^ (*P* < 0.01) and lower SR Ca^2+^ content (*P* < 0.01 or *P* < 0.05) at all three observing time points (3, 10, and 30 days post-Iso daily infusion) (**Figure 3D**), and the Ca^2+^ dyshomeostasis was concurrent with cardiac structural and functional remodeling as shown above. Zacopride pretreatment prevented Iso-induced intracellular calcium overload and the decrease of SR Ca^2+^ content, and the effects were largely reversed by chloroquine (*P* < 0.05 or *P* < 0.01).

#### Western Blotting Demonstrated the Protective Effect of Zacopride on Iso-Induced Cardiomyocyte Ca^2+^ Dyshomeostasis *in Vivo*

**Figure 4:**
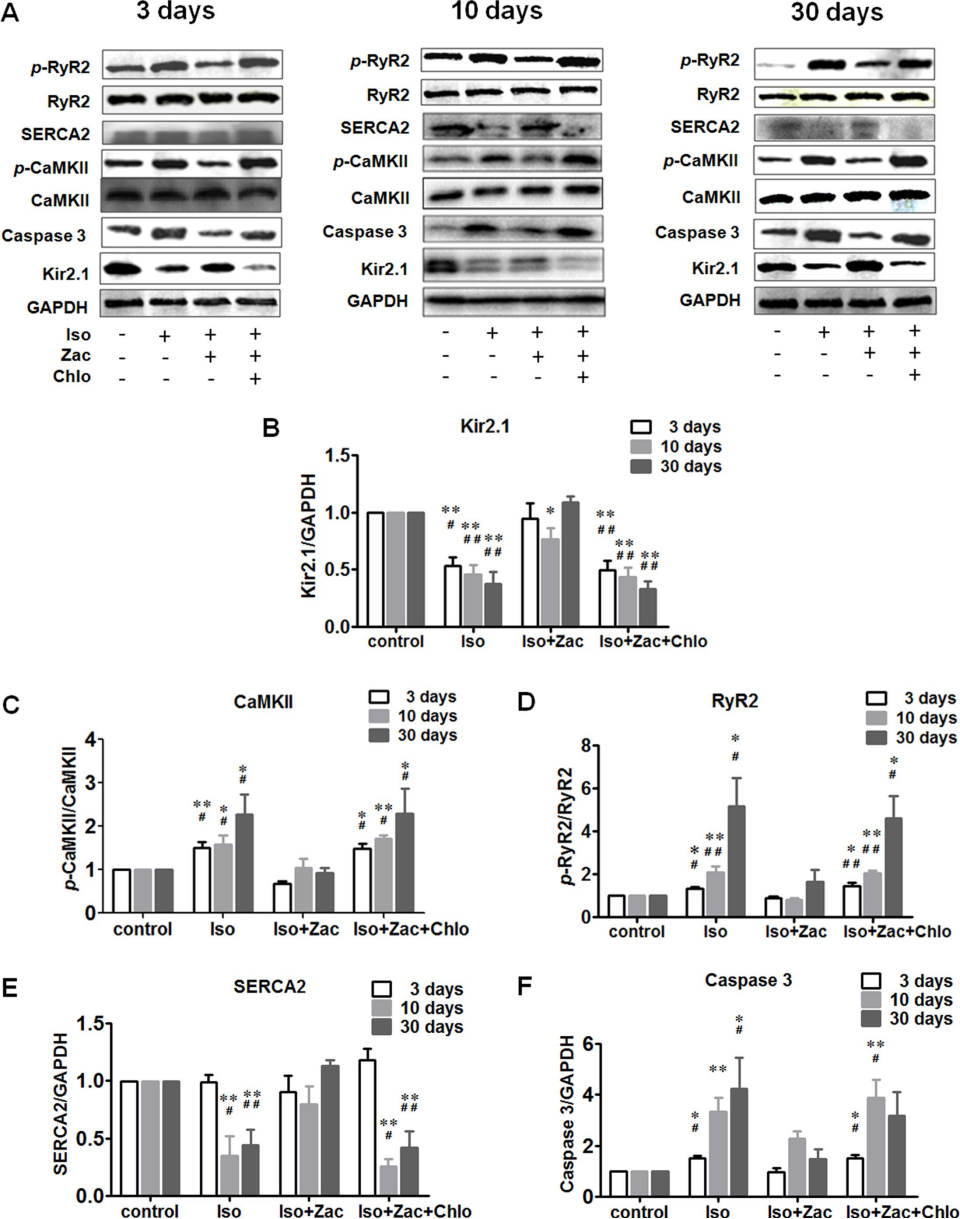
Zacopride improved Iso-induced maladaptive protein alterations in rat hearts. **(A)** Representative western blotting images 3 days, 10 days, and 30 days after the onset of Iso infusion. **(B)** Dynamic changes of Kir2.1 protein expression levels. **(C)** Dynamic changes of *p*-CaMKII protein expression levels relative to total CaMKII. **(D)** Dynamic changes in *p*-RyR2 protein expression levels relative to total RyR2. **(E)** Dynamic changes in SERCA2 protein expression. **(F)** Dynamic changes of cleaved caspase 3 protein expression. All data were normalized to control. Iso, isoproterenol; Zac, zacopride; Chlo, chloroquine. Data were presented as mean ± SEM (n = 3). *P < 0.05, **P < 0.01 compared with control. ^#^*P* < 0.05, ^##^*P* < 0.01 compared with Iso+Zac.

*In vivo* experiments show that treatment with Iso for 3, 10, and 30 days all decreased the expression of Kir2.1 in cardiomyocytes (**Figures 4A, B**) (*P* < 0.01 *vs*. control). Concurrently, the phosphorylated CaMKII (**Figure 4A, C**) and RyR2 protein levels (**Figure 4A, D**) were progressively elevated (*P* < 0.05 or *P* < 0.01 *vs*. control). SERCA 2 protein (**Figure 4A, E**) did not change during short-term (3 days) Iso challenge, but was downregulated in 10 days and 30 days of Iso groups (*P* < 0.01). Iso also activated caspase 3 (**Figure 4A, F**) as indicated by elevation of cleaved caspase 3 (*P* < 0.05 or *P* < 0.01 *vs*. control) along with the progression of cardiac remodeling. These alterations were largely normalized by zacopride treatment (*P* < 0.05 or *P* < 0.01), and lower dose chloroquine reversed the effects of zacopride (*P* < 0.05 or *P* < 0.01).

### Patch Clamp Experiments Showed the Suppressing Effect of Zacopride on Iso-Induced Changes of Transmembrane Potentials in ARVMs *in Vitro*

**Figure 5:**
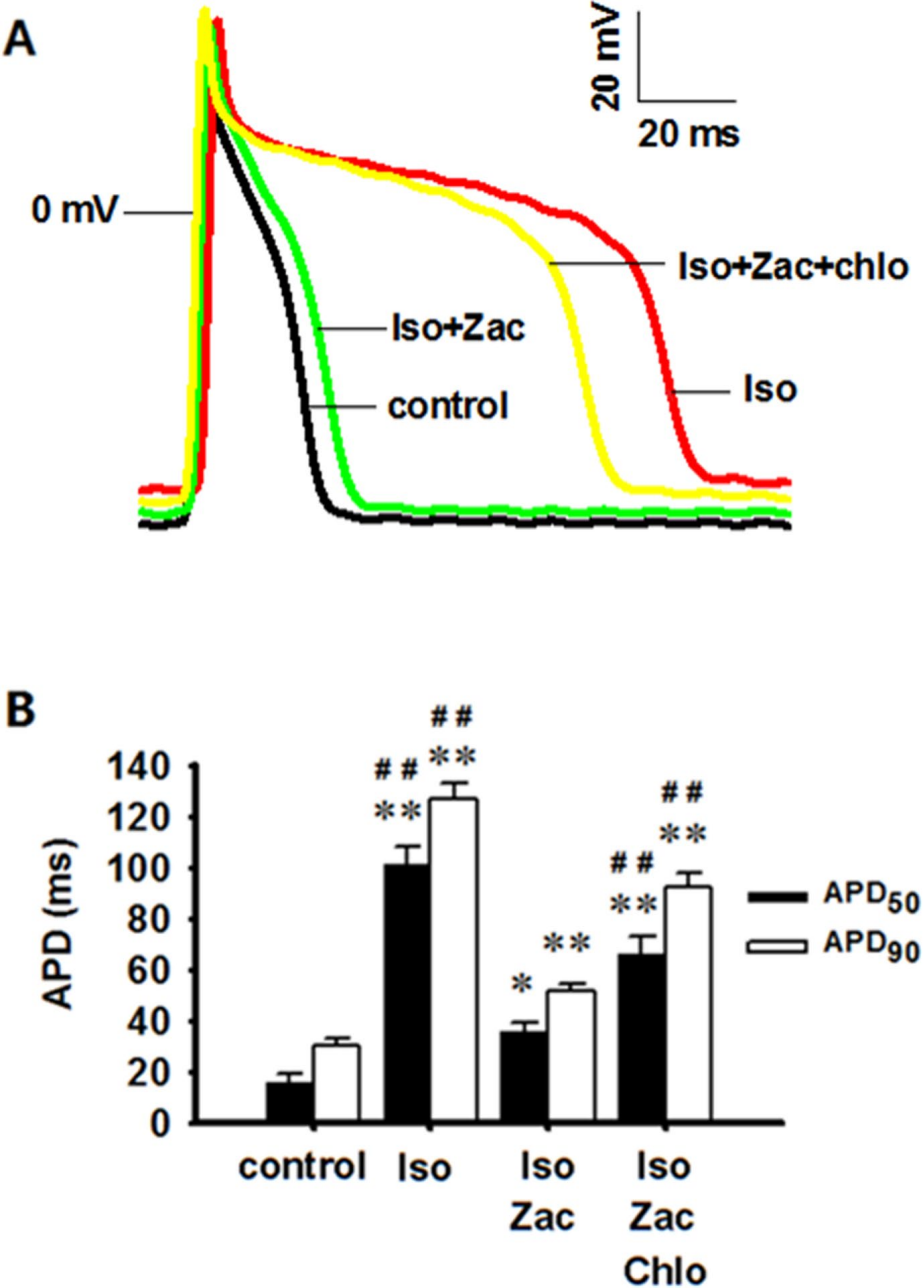
Zacopride restored RP depolarization and APD prolongation in isolated rat ventricular myocytes 10 days post-Iso infusion. These effects could be partially reversed by chloroquine. **(A)** Representative AP recording. **(B)** APD_50_ and APD_90_ in different groups. Iso, isoproterenol; Zac, zacopride; Chlo, chloroquine. N = 6 cells. Data were presented as mean ± SEM. *P < 0.05, **P < 0.01 compared with control. ^##^*P* < 0.01 compared with Iso+Zac.

Iso treatment for 10 days induced significant depolarization of RP (*P* < 0.01 *vs*. control) and prolongations of APD_50_ (*P* < 0.01 *vs*. control) and APD_90_ (*P* < 0.01 *vs*. control). Zacopride treatment restored the RP depolarization (*P* < 0.05) and prolongations of APD_50_ (*P* < 0.01) and APD_90_ (*P* < 0.01) to normal or near normal levels. These effects were largely abolished by chloroquine (*P* < 0.01) (**Figure 5**, **Table 3**).

**Table 3:** The effects of zacopride on the morphology of action potential (AP).

	N	RP (mV)	APA (mV)	APD_50_ (ms)	APD_90_ (ms)
Control	6	–75.2 ± 0.8	105.8 ± 4.4	16.0 ± 3.3	30.1 ± 2.9
Iso	6	–66.1 ± 3.2^**#^	108.7 ± 4.6	101.3 ± 7.2^**##^	127.0 ± 5.8^**##^
Iso+Zac	6	–73.1 ± 2.2	109.2 ± 3.7	35.6 ± 3.3^*^	51.4 ± 3.4^**^
Iso+Zac+Chlo	6	–63.8 ± 2.2^**##^	100.4 ± 3.1	66.1 ± 6.6^**##^	92.3 ± 5.6^**##^

APA, action potential amplitude; APD_50_ and APD_90_, action potential duration at 50% and 90% of repolarization, respectively; RP, resting potential; Iso, isoproterenol; Zac, zacopride; Chlo, chloroquine. *P < 0.05, **P < 0.01 vs. control; ^#^P < 0.05, ^##^P < 0.01 vs. Iso+Zac.

### Zacopride Prevented Iso-Induced Hypertrophy and Intracellular Ca^2+^ Overload in NRVMs *in Vitro*

**Figure 6:**
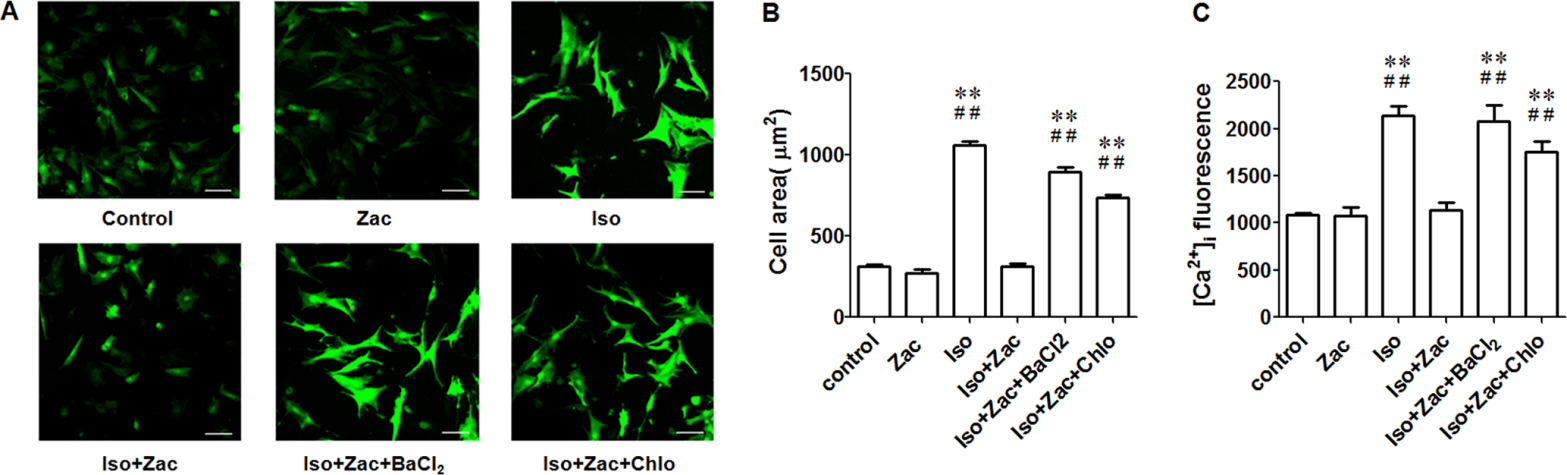
Zacopride inhibited Iso-induced hypertrophy and intracellular calcium overload in cultured NRVMs. **(A)** Fluorescent images of Fluo-4/AM loaded neonatal cardiomyocytes. Bar = 50 μm. **(B)** Zacopride treatment normalized the cell size of NRVMs. **(C)** Zacopride treatment attenuated Iso-induced intracellular calcium overload. BaCl_2_ or chloroquine reversed the effects of zacopride on cell sizes **(B)** and [Ca^2+^]_i_**(C)**. Zacopride had no effect on [Ca^2+^]_i_ in normal NRVMs. Iso, isoproterenol; Zac, zacopride; Chlo, chloroquine. N = 6 cells. Data were presented as mean ± SEM. **P < 0.01 compared with control; ^##^*P* < 0.01 compared with Iso+Zac.

Iso at 1 μmol/L induced hypertrophy of cultured neonatal cardiomyocytes as evidenced by enlarged cell area and higher [Ca^2 +^]_i_ (*P* < 0.01) (**Figure 6**). Zacopride treatment restored cell morphology to normal or near normal levels (**Figure 6B**). This *in vitro* result was consistent with that from ARVMs. Confocal detection indicates that zacopride significantly attenuated Iso-induced calcium overload (*P* < 0.01) (**Figure 6C**). This effect was reversed by I_K1_ channel blockers BaCl_2_ or chloroquine (*P* < 0.01). Notably, zacopride had no effect on [Ca^2 +^]_i_ in normal NRVMs, suggesting that suppression of Ca^2+^ overload by zacopride may contribute to the protective effect of zacopride on cardiac remodeling.

### Zacopride Inhibited Iso-Induced Apoptosis of NRVMs

**Figure 7:**
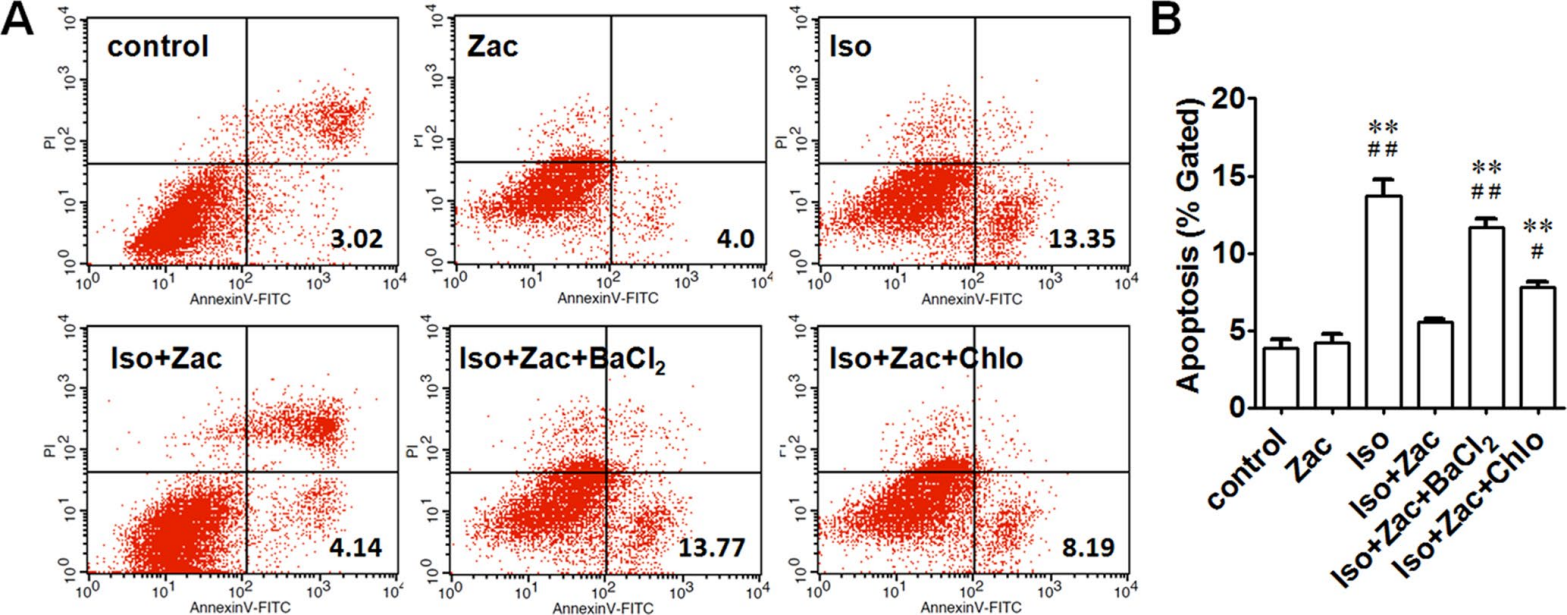
Apoptosis of NRVMs following exposure to 1 μmol/L Iso for 24 h. **(A)** Cells in the lower right (LR) quarter represents early apoptosis. **(B)** Zacopride protected cardiomyocytes from Iso-induced apoptosis. I_K1_ channel blocker BaCl_2_ or chloroquine reversed the effects of Zac. Iso, isoproterenol; Zac, zacopride; Chlo, chloroquine. N = 5 parallel samples in each group. Data were presented as means ± SEM. **P < 0.01, compared with control. ^#^*P* < 0.05, ^##^*P* < 0.01, compared with Iso+Zac.

Annexin V-FITC/PI staining was performed to detect early-stage apoptosis of cells. The apoptosis in Iso-incubated NRVMs was significantly higher than that in controls (13.7 ± 1.0% *vs*. 3.8 ± 0.6%, *P* < 0.01) (**Figure 7**). Zacopride decreased the apoptosis to 5.6 ± 0.2% (*P* < 0.01). I_K1_ channel blocker BaCl_2_ (*P* < 0.01) or chloroquine (*P* < 0.05) reversed the effects of zacopride, indicating that the anti-apoptotic effect of zacopride was mediated by I_K1_ channel activation.

## Discussion

A variety of diseases, including hypertension, coronary heart disease, hereditary defect, and toxic insults may all cause cardiac remodeling. End-stage remodeling is a major contributor in the development of HF. Currently, the principles for improving cardiac remodeling mainly involve the reduction in cardiac workload, improvement of myocardial systolic and diastolic functions, and inhibition of certain gene expression and release of humoral factors that induce cardiac hypertrophy and fibrosis (Hellawell and Margulies, 2012). Although significant progress has been achieved in recent years, the mortality among patients with HF remains high. Therefore, elucidating the molecular mechanisms underlying cardiac remodeling and HF and identifying novel therapeutic strategies to prevent or reverse cardiac remodeling are still important issues in cardiovascular research. The main findings of the present study are that 1) electrical remodeling is concurrent with structural remodeling, and they may not be two independent processes, but are two circumstances of the same scenario. Consequently, reversing electrical disorder might facilitate the improvement of cardiac structure and function; 2) modulation of the function and expression of I_K1_/Kir2.1 channel might be a novel strategy for handling intracellular Ca^2+^; 3) I_K1_ channel is a promising target for the treatment of cardiac remodeling in future clinical interventions.

### Enhancing I_K1_ Prevented Iso-Induced Intracellular Calcium Overload

Ca^2+^ plays pivotal roles in myocardial excitation-contraction coupling, substance metabolism, cell cycle regulation, cell–cell communication, and gene expression (Aiba and Tomaselli, 2010). Intracellular Ca^2+^ homeostasis is maintained by coordination between ATP-dependent ion pumps and transporters located in plasma membranes or organelles, as well as Ca^2+^-binding proteins (Carafoli et al., 2001; Muth et al., 2001). Calcium dyshomeostasis, especially pathologic elevation of intracellular Ca^2+^ (Ca^2+^ overload), is a central event during the development of hypertrophy and HF (Molkentin et al., 1998; Zhang and Brown, 2004). Beta-adrenergic receptor (β-AR) stimulation (such as by Iso) activates the cAMP-dependent kinase (protein kinase A, PKA), consequently leads to the phosphorylation of several Ca^2+^ handling proteins. LTCCs allow Ca^2+^ influx (McDonald et al., 1994), ryanodine receptors (RyRs) are responsible for Ca^2+^ release from sarcoplasmic reticulum (SR), and phospholamban (PLN) reduces inhibition of SR Ca^2+^-ATPase (SERCA) which uptakes cytosolic Ca^2+^. From the view of electrophysiology, depolarized RP and prolonged APD facilitate the opening of LTCCs thus promote intracellular Ca^2+^ accumulation (Swynghedauw, 1999), while I_K1_ downregulation delays AP repolarization and in turn further prolongs APD (Miake et al., 2003). In the ventricle, LTCC is the main Ca^2+^ influx pathway and plays a key role in the excitation-contraction coupling. Overactivation of LTCC elevates intracellular Ca^2+^ and correlates with the genesis of hypertrophy (Muth et al., 2001; Song et al., 2002; Bodi et al., 2003; Viola et al., 2009). LTCC antagonists have been expected to retard Ca^2+^ influx and prevent hypertrophy (Sugiura et al., 2001). However, as Ca^2+^ influx through LTCC is important in initiating and maintaining contraction, LTCC blockers might be limited in the clinical application because of the potential risk of pumping dysfunction.

Elevation of cardiomyocyte [Ca^2+^]_i_ elicits a series of biochemical signals through multifaceted Ca^2+^-regulated enzymes including Ca^2+^-/calmodulin-dependent protein kinase II (CaMKII). Iso is also a definite stimulation for the autophosphorylation of CaMKII in cardiomyocytes (Zhu et al., 2003; Pereira et al., 2007; Mangmool et al., 2010). Activated CaMKII phosphorylates multiple ion channels and Ca^2+^ handling proteins such as voltage-gated Na^+^ channel, LTCC, Cl^−^ channel, and RyR receptor (Grandi et al., 2007; Bers and Grandi, 2009; Wagner et al., 2009). We show here that hyperphosphorylated CaMKII is a key player for intracellular Ca^2+^ dyshomeostasis. Reduction of SR Ca^2+^ content in hypertrophic or failing cardiomyocytes were largely resulted from reduced SERCA2 and increased RyR leak, and in turn, facilitated cytosolic Ca^2+^ overload. If considering the ineffectiveness of zacopride on I_Ca-L_ or I_NCX_ (Liu et al., 2012), the inhibition of Ca^2+^ overload by zacopride is probably secondary and mediated by the activation of I_K1_ channels. By enhancing I_K1_ or upregulating Kir2.1, zacopride reversed RP depolarization and APD prolongation, restored some key Ca^2+^ handling proteins, consequently decreased cardiac Ca^2+^ overload. Of note, zacopride inhibited Ca^2+^ overload in hypertrophic cardiomyocytes but not affected [Ca^2 +^]_i_ in normal cardiomyocytes (**Figure 6**).

### PKA Signaling may be Involved in I_K1_ Channel–Mediated Calcium Homeostasis

Kir2.1, Kir2.2, and Kir2.3 are the substrates of PKA and protein kinase C (PKC). Phosphorylation of these pore-forming channel proteins thus may play important roles in regulating I_K1_ channel function although the mechanism remains a matter of debate (Fakler et al., 1994; Koumi et al., 1995; Sonoyama et al., 2006; Kiesecker et al., 2006). Fakler et al. demonstrate that I_K1_ channels expressed in *Xenopus oocytes* is upregulated by addition of the catalytic subunit of PKA and is downregulated following application of a specific activator of PKC (Fakler et al., 1994). While Koumi et al. found that native I_K1_ channels in guinea-pig ventricular myocytes are inhibited by exposure of the cytosolic side of the membrane to purified catalytic subunit of PKA. In human cardiomyocytes, ET-1 induced a marked inhibition of I_K1_ which could be suppressed by a PKC inhibitor staurosporine but not be altered by PKA inhibitor KT5720 (Kiesecker et al., 2006). In our previous work, zacopride selectively activated heterologously expressed Kir2.1 channels in HEK 293 cells, and the activation could be reversed by PKA inhibitor KT5720 but not by PKC inhibitor GF109203X or PKG inhibitor KT5823. Further mutation of the PKA phosphorylation site S425L completely abolished the agonizing effect of zacopride on Kir2.1. These data suggest that zacopride selectively activates Kir2.1 channel *via* a PKA-mediated signaling pathway (Zhang et al., 2013).

As a heterotetrameric threonine/serine kinase, PKA is also involved in the regulation of intracellular calcium homeostasis. Activated cyclic-AMP/PKA triggers Ca^2+^ influx through LTCC and Ca^2+^ release from SR, resulting in RP depolarization, then Ca^2+^ extrusion from the cardiomyocyte by NCX (Roe et al., 2015). Considering that zacopride has no direct effect on I_NCX_, I_Ca-L_ (Liu et al., 2012), and I_KATP_ in rat LV myocytes (Zhai et al., 2017), enhancing I_K1_ may be an negative feedback which limits the PKA-mediated depolarization and calcium overload.

### The Interplay Between Electrical and Structural Remodelings Around Calcium Overload

In present study, chronic exposure to β-AR agonist Iso induced both structural and electrical remodelings in the rat ventricles. The structural remodeling manifested with increase of ventricle mass, cardiac hypertrophy, apoptosis, abnormalities of proteins expression, and interstitial fibrosis. The electrical remodeling is featured with depolarization of RP, prolongation of APD, downregulation of I_K1_ (Kir2.1) channel, elevation of cytosolic free Ca^2+^, and reduction of SR Ca^2+^ content. Among these events, Ca^2+^ overload is a key factor linking the electrical remodeling and structural remodeling. For example, secondary to Ca^2+^ overload, Ca^2+^-activated CaMKII phosphorylates various transcription factors such as class II HDACs (Backs et al., 2006; Zhang et al., 2007), transcription factor 1(ATF-1) (Sun et al., 1996), cAMP response element–binding protein (CREB) (Sheng et al., 1991), and myocyte enhancer factor 2 (MEF2) (Passier et al., 2000; Zhang et al., 2007). These events promote the expression of cardiac specific genes involved in the structural remodeling. Chronic CaMKII overexpression also caused downregulation of I_K1_ channels (Wagner et al., 2009), whereas inhibition of CaMKII increased I_K1_ density which partially accounted for the shortening of APD (Li et al., 2006). These findings agree well with the observations in the present study albeit the disputable causality. From the *in vivo* time-course study, cardiac electrical remodeling, such as inhibition of I_K1_ and Ca^2+^ dyshomeostasis, is concurrent with the structural remodeling and dysfunction. This connection was also supported by the genesis of cardiac apoptosis. An inappropriate rise in intracellular Ca^2+^ activates Ca^2+^-/Mg^2+^-dependent endonucleases and glutamine transferases which degrade DNA and cytoskeletal proteins. Apoptosis may lead to cardiac fibrosis (Zhang et al., 2011). Therefore, reduction of intracellular calcium overload has been an important focus in the prevention of maladaptive cardiac remodeling. By upregulating cardiac I_K1_, zacopride prevented Iso-induced electrical remodeling, preserved Ca^2+^ homeostasis, and thus inhibited calcium-activated remodeling signaling and apoptosis, thereafter improved structural remodeling. The most convincing data shown here were that BaCl_2_ *in vitro* or chloroquine *in vitro/vivo* could blunt the beneficial effects of zacopride on both electrical and structural remodelings. I_K1_ channel might be a novel target for the regulation of cardiac calcium homeostasis and remodeling.

It is worth mentioning that in the *in vivo* experiment, about 50% rats suffered from sudden death in the follow-up period after Iso infusion. There are two major causes of death in HF: pumping function decline and arrhythmias. These two causes both link to Ca^2+^ dyshomeostasis in cardiomyocytes (Bers, 2006). Cardiac pumping dysfunction may not sufficiently account for the sudden death in the present study, because the rats subjected to 3 days and 10 days of Iso infusion showed enhanced pumping function. Acute Iso challenging induced DADs which facilitate arrhythmogenesis (Zhai et al., 2017). Ventricular tachyarrhythmia and LV hypertrophy both increase the risk of sudden cardiac death (SCD) up to 10-fold (Aronow et al., 1988), and resolution of LV hypertrophy reduces the risk of SCD (Wachtell et al., 2007). As an I_K1_ channel agonist, zacopride has been recognized as a new antiarrhythmic agent on triggered arrhythmias (Liu et al., 2012; Zhai et al., 2017). Here, we further show that zacopride was also effective in reducing cardiac hypertrophy. The dual actions of zacopride might protect animals from SCD. We expect that targeting myocardial I_K1_ channel and Ca^2+^ homeostasis may have great potential for the prevention of triggered arrhythmias in HF patients.

## Data Availability

All datasets generated for this study are included in the manuscript and the supplementary files.

## Ethics Statement

This study was carried out in accordance with the recommendations of the guidelines for the Care and Use of Laboratory Animals (NIH, revised 2011), Ethics Committee of Shanxi Medical University. The protocol was approved by the Ethics Committee of Shanxi Medical University.

## Author Contributions

Q-HL, designed the study, performed the experiments, and drafted the manuscript. XQ, L-JZ, YL, X-NC and X-ZR acquired and analyzed the data. Q-LF, analyzed the data. JW performed studies evaluating cardiac function. LZ and X-WZ carried out the patch clamp experiments. B-WW and J-MC participated in the protocol design and critically revised the manuscript, and gave the final proof for the manuscript.

## Funding

This work was supported by grants from the National Natural Science Foundation of China (No. 31200864 to Q-HL, No. 81670313 to J-MC), a grant from Shanxi Scholarship Council of China (No. 2016-059 to Q-HL), grants from the Key Laboratory of Medical Electrophysiology (Southwest Medical University), Ministry of Education of China (No. 201704 to Q-HL), and a fund for Shanxi “1331 Project” Key Subjects Construction (1331KSC).

## Conflict of Interest Statement

The authors declare that the research was conducted in the absence of any commercial or financial relationships that could be construed as a potential conflict of interest.

## Abbreviations

APD, action potential duration; I_K1_, inward rectifier potassium channel or current; ARVM, adult rat ventricular myocyte; NRVM, neonatal rat ventricular myocyte; Iso, isoproterenol; Chlo, chloroquine; Zac, zacopride; LTCC/I_Ca-L_, L-type calcium channel; I_to_, transient outward K^+^ current; I_KATP_, ATP-sensitive potassium current; I_NCX_, Na^+^-Ca^2+^ exchanger (NCX) current; RP, resting potential; SERCA, sarcoplasmic reticulum Ca^2+^-ATPase; CaMKII, Ca^2+^-/calmodulin-dependent protein kinase II; DAD, delayed afterdepolarization; *m*-CPBG, *m*-chlorophenylbiguanide.
