# Supplementary material for: IK1 Channel Agonist Zacopride Alleviates Cardiac Hypertrophy and Failure via Alterations in Calcium Dyshomeostasis and Electrical Remodeling in Rats
Source: Front Pharmacol. 2019 Aug 26;10:929. doi: 10.3389/fphar.2019.00929 (PMC6718093; doi:10.3389/fphar.2019.00929)
Supplement: Supplementary file 1 [file Table_1.docx]

Table S1. Informations about the grouping and rat numbers included in

each experiment in vivo.

|  | **Echo**  **(n)** | **Heart weight (n)** | **Histology**  **(n)** | **Confocal**  **(n)** | **WB**  **(n)** | **Patch**  **clamp (n)** |
| --- | --- | --- | --- | --- | --- | --- |
| **3 days** |  |  |  |  |  |  |
| control | 5 |  |  | 2 | 3 |  |
| Iso | 5 |  |  | 3 | 3 |  |
| Iso+Zac | 5 |  |  | 3 | 3 |  |
| Iso+Zac+Chlo | 5 |  |  | 3 | 3 |  |
| **10 days** |  |  |  |  |  |  |
| control | 6 | 9 | 5 | 3 | 6 | 2 |
| Zac | 6 | 11 | 5 |  |  |  |
| Chlo | 6 | 9 | 5 |  |  |  |
| Iso | 7 | 9 | 5 | 3 | 6 | 2 |
| Iso+Zac | 6 | 11 | 5 | 3 | 6 | 2 |
| Iso+Chlo | 5 | 8 | 5 |  |  |  |
| Iso+Zac+Chlo | 6 | 10 | 5 | 3 | 6 | 2 |
| Iso+Zac+RS23597 | 5 | 5 | 3 |  |  |  |
| Iso+Zac+*m*-CPBG | 5 | 5 | 3 | 2 |  |  |
| **30 days** |  |  |  |  |  |  |
| control | 6 |  |  | 3 | 3 |  |
| Iso | 6 |  |  | 3 | 3 |  |
| Iso+Zac | 7 |  |  | 3 | 3 |  |
| Iso+Zac+Chlo | 5 |  |  | 2 | 3 |  |
